# Enhancing and inhibiting stimulated Brillouin scattering in photonic integrated circuits

**DOI:** 10.1038/ncomms7396

**Published:** 2015-03-04

**Authors:** Moritz Merklein, Irina V. Kabakova, Thomas F. S. Büttner, Duk-Yong Choi, Barry Luther-Davies, Stephen J. Madden, Benjamin J. Eggleton

**Affiliations:** 1 Centre for Ultrahigh bandwidth Devices for Optical Systems (CUDOS), Institute of Photonics and Optical Science (IPOS), School of Physics, University of Sydney, Sydney, New South Wales 2006, Australia; 2 CUDOS, Laser Physics Centre, Research School of Physics & Engineering, Australian National University, Canberra, Australian Capital Territory 0200, Australia

## Abstract

On-chip nonlinear optics is a thriving research field, which creates transformative opportunities for manipulating classical or quantum signals in small-footprint integrated devices. Since the length scales are short, nonlinear interactions need to be enhanced by exploiting materials with large nonlinearity in combination with high-Q resonators or slow-light structures. This, however, often results in simultaneous enhancement of competing nonlinear processes, which limit the efficiency and can cause signal distortion. Here, we exploit the frequency dependence of the optical density-of-states near the edge of a photonic bandgap to selectively enhance or inhibit nonlinear interactions on a chip. We demonstrate this concept for one of the strongest nonlinear effects, stimulated Brillouin scattering using a narrow-band one-dimensional photonic bandgap structure: a Bragg grating. The stimulated Brillouin scattering enhancement enables the generation of a 15-line Brillouin frequency comb. In the inhibition case, we achieve stimulated Brillouin scattering free operation at a power level twice the threshold.

## Abstract (web summary)

On-chip nonlinear optics can be used to manipulate classical or quantum signals but enhancement of competing nonlinear processes can cause signal distortion. Here, Merklein *et al*. enhance and inhibit nonlinear scattering on a chip by tailoring the optical density-of-states at the edge of a photonic bandgap.

## Graphical abstract

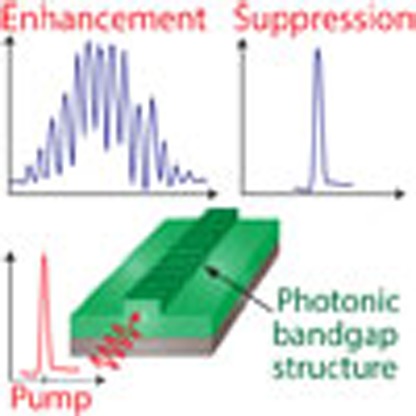

Enhanced nonlinear interactions and spontaneous emission near the band edge of periodic structures^1^,^2^ have fascinated researchers for decades as a possible path towards power-efficient integrated photonics. The combination of strong field enhancement with prolonged light–matter interaction due to slow-light propagation^3^,^4^,^5^ is a powerful technique for overcoming material constraints and can lead to a significant reduction in the size of photonic structures. On the basis of this idea, several milestone demonstrations^6^,^7^,^8^,^9^ in photonic circuits have been reported, including slow-light enhanced third-harmonic generation^10^, four-wave mixing (4WM)^11^, slow-travelling solitons^12^ and gain enhancement in lasers^13^. Surprisingly, band-edge effects on stimulated Brillouin scattering (SBS), one of the strongest nonlinear phenomena, have not been experimentally demonstrated to date. A power-efficient way to generate SBS, however, would advance on-chip SBS applications, in particular Brillouin lasers^14^ and Brillouin frequency combs^15^.

From another point of view, nonlinear interactions can limit the power efficiency in optical fibre communication links^16^. SBS, for example, is known to be detrimental for high-power continuous wave (CW) lasers and amplifiers^16^,^17^,^18^ and degrades the performance of fibre and chip-based signal processors that exploit the Kerr nonlinearity^16^. In particular, the suppression of SBS in CW-pumped signal processing devices remains a significant challenge, calling for specially designed fibres with reduced Brillouin gain^16^. Similar problems arise in the context of on-chip phase-sensitive amplifiers and regenerators, which also rely on CW pumping^19^,^20^. The ability to fully suppress SBS will therefore remove power limitations and lead to more efficient nonlinear interactions.

Current approaches minimize the generation of SBS by increasing the pump bandwidth via modulation of the pump phase and amplitude^19^, or by using structures with poor acoustic guidance and hence low SBS gain such as silicon on insulator waveguides^20^. Nonlinear signal processing in silicon waveguides at telecommunication wavelengths has, however, its own limitations due to the presence of nonlinear absorption and free carrier generation^21^,^22^. Here we present an experimental demonstration of an alternative approach, in which the efficiency of SBS is tailored by the presence of a photonic bandgap (PBG).

The combination of SBS and a Bragg grating was previously studied in numerical models in the context of nonlinear pulse propagation^23^ and improved delay line performance^24^. The latter study proposed a linear sequence of SBS-based slow-light and grating-based slow-light to increase the system’s overall delay^24^. On the other hand, the use of a PBG structure has been suggested to suppress SBS inside the bandgap^25^. This concept is quite similar to the previously explored inhibition of spontaneous emission inside the bandgap of photonic crystals^1^,^26^. The experimental demonstration of SBS inhibition, however, remains challenging because the bandwidth of the periodic structure must be narrow enough to suppress the Stokes signal without impacting the pump, and additionally the length of the periodic structure must match the length of the SBS gain medium. Therefore, 1-m-long Bragg gratings were proposed to suppress SBS in silica fibres in the context of Q-switched lasers^25^, whereas 4WM-based optical signal processing architectures usually use hundreds of metres of highly nonlinear fibre^16^ and would require prohibitively long periodic structures.

The length of the SBS gain medium can be reduced to only a few centimeters using highly nonlinear materials to create photonic integrated circuits^27^. For example, very large Brillouin gain *G*_SBS_ can be achieved in an As_2_S_3_ chalcogenide waveguide (gain parameter *g*_0_=0.715 × 10^−9^ m W^−1^)^28^ due to the small effective mode area, *A*_eff_, combined with high material refractive index and excellent overlap between optical and acoustical modes^28^,^29^. Thus the Brillouin gain *G*_SBS_ can be about 500 times larger than in a silica single-mode fibre^30^. The large value of *G*_SBS_ in such chalcogenide rib waveguides led to the first demonstration of on-chip SBS^28^.

In this article, we demonstrate both enhancement and inhibition of nonlinear scattering in a photonic integrated circuit by tailoring the optical density-of-states (DOS) associated with a one-dimensional PBG, a stopband of a Bragg grating, written into a chalcogenide rib waveguide. We exploit slow-light enhancement of SBS to demonstrate a 15-line Brillouin frequency comb that is at least five Stokes orders broader than in demonstrations using chalcogenide waveguides reported previously^31^. In addition, we exploit the inhibition of nonlinear scattering inside the stopband to suppress SBS and achieve linear transmission at higher powers. Both demonstrations illustrate a unique capacity for tailoring the strength of a nonlinear interaction on a chip solely by means of frequency tuning. Waveguide-long Bragg gratings in chalcogenide photonic chips can be written using interferometric techniques^32^ or internal inscription^33^,^34^ and these enable both regimes, SBS enhancement and inhibition. The approach of using Bragg gratings provides several advantages over high-index contrast photonic crystals for the enhancement and inhibition of SBS, namely the narrow bandwidth of the stopband, low loss and potential tunability. The narrow bandwidth is crucial for the inhibition of SBS as only the Stokes wave should be affected by the stopband, while the pump wave will have full transmission. Although we demonstrate tailoring the strength of SBS in a chalcogenide chip, the same concept can be extended to other materials and nonlinear processes, for example, 4WM and Raman scattering.

## Results

### Basic principle

**Figure 1:**
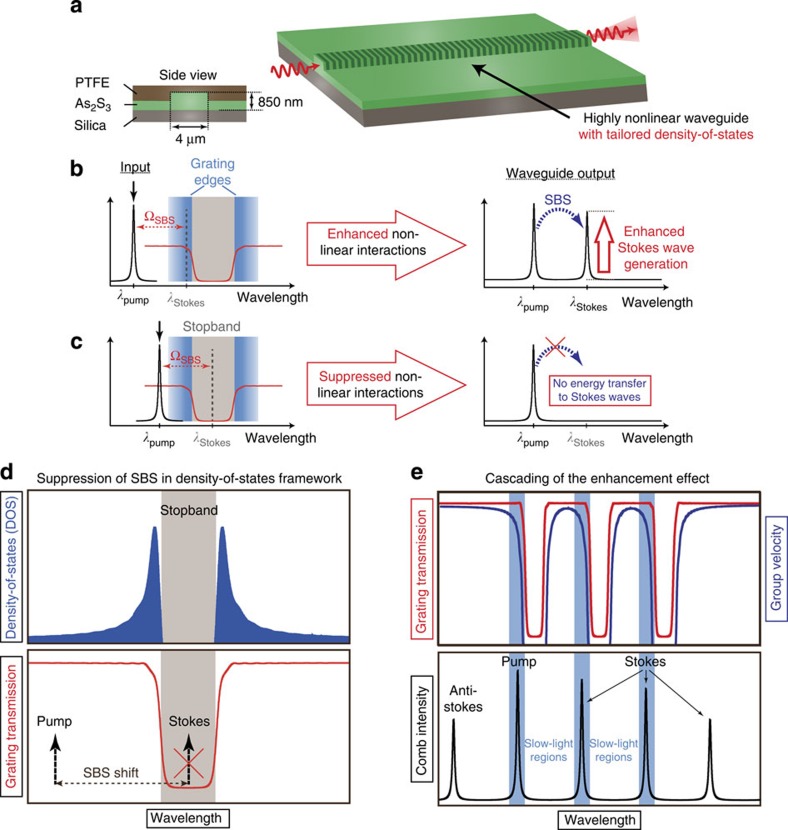
Basic principle for enhancing or inhibiting SBS by tailoring the DOS in a waveguide. (**a**) Scheme of the 850 nm by a 4 μm chalcogenide (As_2_S_3_) rib waveguide on a silica substrate with a polytetrafluoroethylene (PTFE) over-cladding. (**b**) Tuning the frequency of the optical waves (here shown for the 1st Stokes wave) into the slow-light region at the band edge of the PBG leads to an enhancement of the nonlinear interactions. (**c**) Adjusting the pump frequency so that the Stokes frequency falls into the stopband, however, leads to an inhibition of the SBS process. (**d**) Illustration of the distribution of the density-of-states and the stopband for a one-dimensional PBG structure. The DOS increases towards the band edge of the structure and approaches zero in the stopband, which enabled the full suppression of any Stokes wave generation. (**e**) Scheme of how the enhancement effect can be cascaded using a multiband grating to form a Brillouin frequency comb. It illustrates the distinctive design of the multiband grating, matching the Brillouin shift and each having narrow bandwidth, so that multiple comb lines are aligned with the grating edges.

Fig. 1a–c illustrates the concept of enhancing and inhibiting SBS by a structure with a stopband in a highly nonlinear waveguide. A single-frequency laser is sent into a chalcogenide rib waveguide. If the pump power exceeds the threshold^35^ for Brillouin scattering (*P*_SBS_), a backscattered Stokes wave can be observed. The Stokes wave arises from the coherent backscattering of the pump wave from thermally excited acoustic phonons^35^. The phonons are moving along the waveguide at the speed of sound leading, via the Doppler effect, to a frequency downshift of the backscattered Stokes wave. This frequency shift equals the acoustic phonon frequency *Ω*_B_ and is typically in the gigahertz range (*Ω*_B_≈7.7 GHz for As_2_S_3_ at *λ*=1,550 nm).

Integrating a PBG structure in the gain medium gives an opportunity to tailor the nonlinear interaction strength, enabling both, enhancement and suppression of SBS. This concept is illustrated in Fig. 1b,c. Adjusting the pump wavelength so that the Stokes wavelength coincides with the edge of the PBG leads to an enhancement of the Stokes wave generation (Fig. 1b), whereas it is inhibited when the Stokes wavelength falls into the stopband (Fig. 1c). For wavelengths inside the stopband, light is not allowed to propagate, the transmission reaches a minimum and hence no field can build up at these wavelengths. Near the band edges, however, a light signal travels on average slower due to multiple coherent reflections from the refractive index modulation, leading to a field enhancement. This is similar to the description of electron behaviour near the electronic bandgap and the DOS formulation in solid-state physics^1^,^36^,^37^. Using this analogy, the optical DOS is expected to change drastically in the vicinity of the photonic stopband: at the band edge, in the slow-light region, the DOS increases, while it approaches zero inside the stopband (Fig. 1d). We exploit the low DOS inside the stopband to inhibit the build-up of a Stokes wave and suppress SBS. On the other hand, the high DOS at the edge of the stopband can be used to enhance the generation of a Stokes wave.

If the power of the 1st Stokes wave exceeds *P*_SBS_, SBS can be cascaded generating a 2nd-order Stokes wave shifted by 2*Ω*_B_ from the pump. The power requirements for cascaded SBS can be reduced substantially in resonators^31^,^38^,^39^. For the rib waveguides we use, 17% reflectivity is expected from the cleaved facets due to the large index mismatch between As_2_S_2_ (≈2.43) and the surrounding air. The combination of high SBS gain material and feedback from the facets already enabled the observation of a 2nd-order Stokes wave in a chip-scale device^31^. However, for practical applications in microwave photonics and arbitrary waveform generation, on-chip sources with a larger number of spectral lines are necessary, thus the efficiency of the SBS cascading process has to be further improved.

PBG structures can be a solution for power-efficient SBS cascading and the formation of broad-bandwidth Brillouin combs. This, however, requires a distinctive design of the PBG structure, featuring multiple stopbands exactly matching the Brillouin shift *Ω*_B_ and being narrow bandwidth. A schematic of such a structure is shown in Fig. 1e, which illustrates the alignment of the pump and several Stokes waves with the edges of the multiple stopbands, leading to simultaneous enhancement of several waves.

### Enhancement of SBS

**Figure 2:**
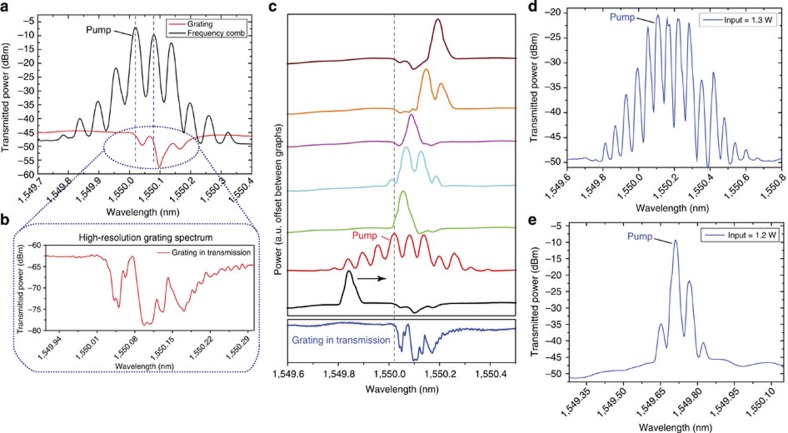
Slow-light enhancement effect of stimulated Brillouin scattering. (**a**) Alignment of the Brillouin frequency comb with respect to the multi-stopband Bragg grating. The pump and lowest-order Stokes waves lay at the edges of the individual stopbands, where the DOS was high but the normalized transmission was almost unity. (**b**) High-resolution spectrum of the multi-stopband structure. (**c**) Wavelength scan across the grating spectrum. The SBS process was significantly enhanced in the slow-light regions. (**d**) Brillouin frequency comb with 15 lines generated by harnessing the tailored DOS in the waveguide. (**e**) Maximum SBS cascading in the waveguide without the grating.

First we demonstrate the enhancement of the SBS cascading process near the band edge of a grating. A measured spectrum of a Brillouin frequency comb generated using this method is shown in Fig. 2a. The graph shows the output comb spectrum in transmission and the alignment of the comb lines with the edges of the multiple bands of the grating (red spectra in Fig. 2a). The superstructure grating was inscribed into the highly nonlinear rib waveguide beforehand, following the method discussed in ref. 34. The three stopbands of this grating were formed by designing the writing beam to be a cascade of three waves (pump, 1st Stokes and 2nd Stokes). This ensured the separation between the gaps to be exactly the frequency shift *Ω*_B_ in the chalcogenide waveguide (≈64 pm)^28^. A high-resolution spectrum of our multiband grating is shown in Fig. 2b.

The enhancement effect at the band edges of the grating was demonstrated by scanning the pump laser wavelength across the grating spectrum (Fig. 2c), with the pump power (*P*_P_≈0.5 W) adjusted just below the measured power threshold *P*_SBS_≈0.6 W. The measured value for *P*_SBS_ agrees well with the theoretically calculated threshold given by^40^:


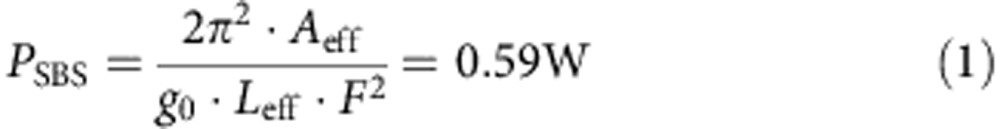


with the SBS gain parameter, *g*_0_=0.715 × 10^−9^ m W^−1^, the effective length *L*_eff_=5.5 cm, *A*_eff_=2.3 μm^2^ and the measured finesse of the cavity *F*=1.40. The pump power *P*_P_ was kept constant during the wavelength sweep and no Stokes waves were generated at frequencies far from the photonic stopband (black curve in Fig. 2c). However, aligning the pump wavelength with the first band edge of the multiband structure led to a strong enhancement of the SBS generation and cascading process (red curve in Fig. 2c). This enabled the formation of a Brillouin frequency comb with four Stokes waves (1S–4S) at a sub-threshold (≤*P*_SBS_) power level. Tuning the pump further towards the longer wavelength showed an enhancement effect, although more moderate, every time the pump wave hit one of the band edges of the multiband grating.

The largest enhancement effect is observed when the waves with the highest intensities in the waveguide, namely the initial pump, 1st and 2nd Stokes wave coincide with the band edges of the grating. A Brillouin comb with 15 distinct comb lines could be generated by increasing the pump power to about 1.3 W≈2 × *P*_SBS_ (Fig. 2d). This is the broadest Brillouin frequency comb generated in a chip-scale low-Q waveguide, exceeding previous demonstrations by five Stokes orders^31^. Recently Büttner *et al*.^15^ demonstrated that Brillouin frequency combs generated in a low-finesse chalcogenide fibre cavity show phase locking mediated by an interplay of 4WM and SBS. This is an important finding since phase locking is crucial for all frequency comb applications. The enhancement effect demonstrated in this article provides a way to increase the effective SBS gain in a highly nonlinear waveguide and shows a path towards power-efficient, on-chip integrated GHz repetition rate light sources.

**Figure 3:**
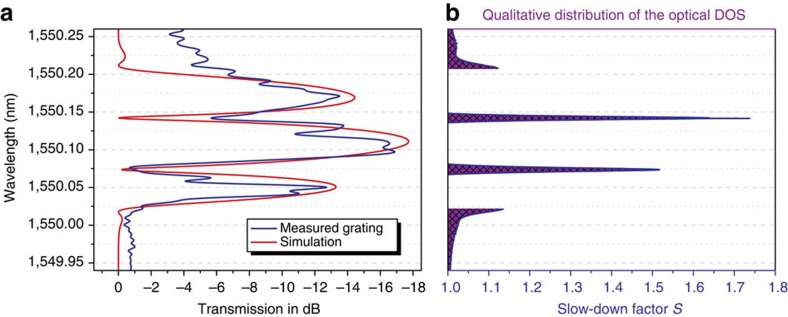
Simulation of the PBG structure and the group index. (**a**) High-resolution grating spectrum and simulated grating spectrum obtained by solving the coupled equations for the transmitted and reflected waves in the waveguide. (**b**) Calculated slow-down factor *S* from the simulated grating. The graph also represents qualitatively the distribution of the DOS in the waveguide.

The enhancement of the nonlinear interactions at the band edge of the PBG can be attributed to an amplified light–matter interaction in the slow-light regime by a factor *S*=*n*_*g*_/*n*. This slow-down factor *S* determines how much slower a light signal travels in the PBG structure compared with the bare waveguide. Figure 3a shows a measured (blue) and simulated (red) transmission spectrum, respectively, for the multi-wavelength Bragg grating used in our experiment. The red spectrum was obtained by solving the coupled-mode equations and presents a good fit to the experimental data. On the basis of the numerical fit, we can determine the slow-down factor *S* for each frequency across the grating spectrum (Fig. 3b). The slow-down factor *S* can be deduced by normalizing the group delay^24^ 
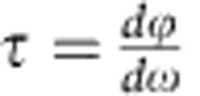
 that a light signal acquires during propagation through the Bragg grating to the transit time *τ*_tr_=550 ps in the waveguide without grating (*ϕ* describes the phase transfer function of the optical wave). We find that the slow-down factor reaches its maximum value of *S*=1.75 at the band edge of the second stopband (Fig. 3b). It is well known for photonic crystals that the optical DOS is proportional to *S* at the band edges^41^ and approaches zero in the stopband. Figure 3b gives a qualitative distribution of the optical DOS in the vicinity of the PBG structure.

The SBS gain *G*_SBS_ increases exponentially with the Brillouin gain coefficient *g*_0_ and the input pump power *P*_P_ (ref. 35). In a similar approach as used to describe the gain enhancement of band-edge lasers^13^, *G*_SBS_ is expected to increase in the slow-light region due to a build-up of the local energy density proportional to *S* (ref. 42). The energy build-up is a result of the coherent coupling of forward- and backward-travelling waves through the grating. However, with *S*<2 this approach cannot fully explain the significant enhancement in the SBS cascading process we observe in our experiments. Below we discuss additional factors that influence the comb formation and determine the power efficiency of this process.

First, the high optical DOS at frequencies near the grating band edges leads to enhanced spontaneous scattering at these frequencies by *S* (refs 43, 44). As shown by Smith^45^, the Stokes power is given by the sum of all spontaneously scattered photons along the gain medium multiplied by the Brillouin gain. Thus, an increase in the optical DOS by a factor of *S* will lead to *S*-times higher Stokes power, in accordance with Fermi’s golden rule, which follows directly from the second-order perturbation theory^46^.

Second, chalcogenide glass is a material with strong *χ*^3^ nonlinearity as well as Brillouin gain. This implies strong multi-wave interactions between the comb components. As soon as the 1st Stokes wave is generated at frequency *ω*_1S_=*ω*_p_−*Ω*_B_ and is reflected by the waveguide facet, it co-propagates with the pump leading to 4WM and the generation of a signal at the frequency *ω*_2S_=*ω*_p_−2*Ω*_B_ and an idler at the frequency *ω*_1AS_=*ω*_p_+*Ω*_B_. These, in turn, can excite higher-order waves at frequencies *ω*_nS_=*ω*_p_−*nΩ*_B_ and *ω*_nAS_=*ω*_p_+*nΩ*_B_. In this manner generated optical waves act as seeds for the SBS process and, therefore, have the ability to reinforce SBS and lower the threshold for the generation of higher-order Stokes waves. Previous studies demonstrated a 10-fold reduction of the SBS threshold when the Stokes wave was seeded instead of it being initiated by noise^47^,^48^,^49^. It is worth noting that for a given power (*P*), the 4WM gain in the chalcogenide rib waveguides is at least 30 times lower than the SBS gain. If we assume zero phase mismatch (largest gain) for the 4WM process, we obtain the following expression for the 4WM and SBS gain coefficients, respectively^50^:


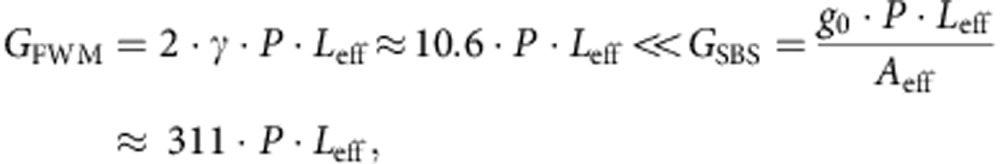


with the nonlinear parameter *γ*. However, the strength of 4WM interactions that include comb components aligned with the band edges of the PBG structure increases^11^ since the intensities of these components are enhanced due to a coherent energy build-up. The large amount of anti-Stokes components of the Brillouin frequency comb in Fig. 2 is a strong indicator that 4WM is present in the comb formation. However, the asymmetry of the comb spectrum demonstrates that SBS is the dominant process.

### Inhibition of SBS

**Figure 4:**
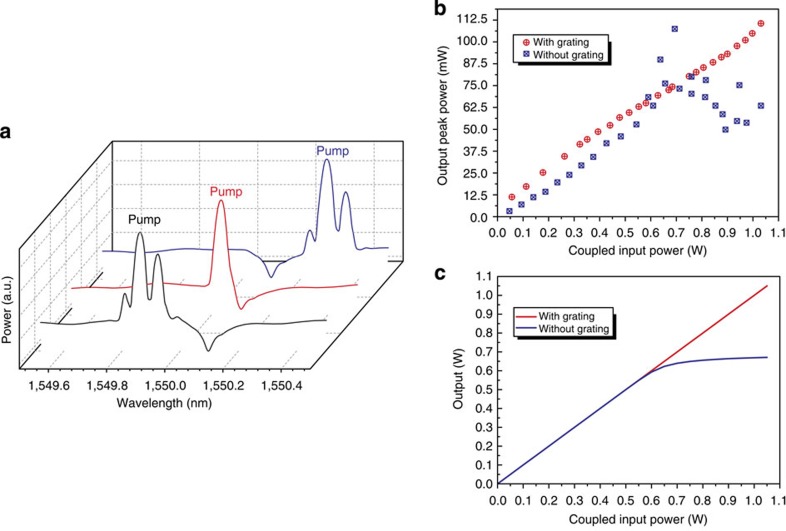
Harnessing the tailored DOS to fundamentally inhibit SBS in the waveguide. (**a**) Spectra obtained as the wavelength of the pump (*P*_p_=0.8 W) was scanned relative to the PBG structure. The power was just above the SBS threshold *P*_SBS_=0.6 W and a Stokes and anti-Stokes wave was generated at frequencies outside the stopband. By positioning the pump so that the Stokes wave fell into the stopband, SBS was no longer generated. (**b**) Pump power output with (red data points) and without (blue data points) the PBG structure. Without the grating, the pump was depleted as soon as the SBS threshold was reached, while with the grating the generation of SBS was inhibited and no pump depletion was observed. (**c**) Simulations of the experiment obtained by solving coupled-mode equations with and without grating confirming the measurements presented in **b**.

As we have already noted, the wavelength-dependent DOS near the band edge of a periodic structure allows not only the enhancement of a nonlinear interaction but also its suppression. It is, therefore, possible to suppress spontaneous and stimulated Brillouin scattering completely if the frequency of the Stokes wave falls into the stopband. The pump wave, however, can lie outside the bandgap because it is shifted by the Brillouin frequency, *Ω*_B_. Provided the bandwidth of the bandgap is narrower than *Ω*_B_, the propagation of the pump is unaffected by the periodic structure. Since no Stokes wave is generated in this situation, the transmission of the pump wave remains linear at any power and pump depletion does not occur. Figure 4a demonstrates this concept using a single stopband Bragg grating. The pump wavelength was tuned across the stopband, whereas the pump power was fixed above the SBS threshold *P*_p_=0.8 W>*P*_SBS_. As can be seen in Fig. 4a, away from the grating stopband a strong Stokes wave was generated. The Stokes wave, however, was suppressed when the wavelength of the Stokes wave *λ*_S_ coincided with the centre of the grating stopband *λ*_B_. To quantify the inhibition of the Stokes wave, we measured the output pump power for different input power levels with and without grating (Fig. 4b). A narrow bandpass filter with 80 pm bandwidth was used before the detector to isolate the transmitted pump power from the Stokes power. For the Stokes wavelength *λ*_S_≠*λ*_B_, SBS generation occurred and the pump power was depleted at power levels above the SBS threshold (blue squares in Fig. 4b). This, however, does not happen when *λ*_S_=*λ*_B_ and therefore the transmitted pump power grew linearly with the input power and no sign of depletion could be seen (red circles in Fig. 4b).

We modelled our system using coupled-mode theory, similarly to that in ref. 25. The results of the numerical modelling are shown in Fig. 4c and show excellent agreement with the experiment. This demonstration of SBS suppression can have a significant impact on the efficiency of ultrafast chip-based all-optical signal processing^51^,^52^. We note that complete SBS suppression for an arbitrary input pump power is only possible in theory for infinitely strong gratings. In practice, however, it is not required and doubling or tripling the SBS threshold can be substantial.

**Figure 5:**
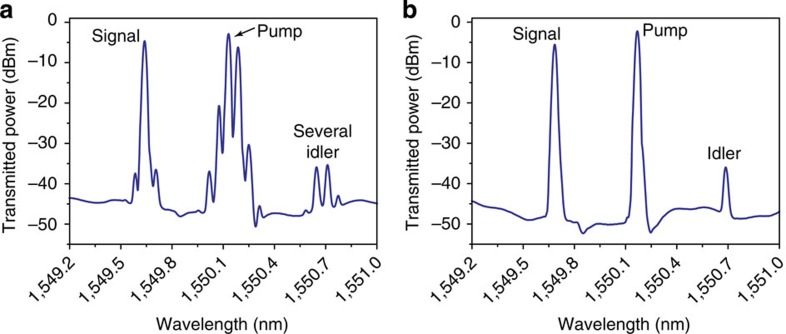
Utilizing the tailored DOS to inhibit SBS in a four-wave mixing experiment. (**a**) Four-wave mixing experiment without the Bragg grating. SBS generated several idlers and, therefore, limited the maximum input power and could lead to parasitic beat signals between several SBS lines. (**b**) The same experiment in the presence of the grating. For the same input power no SBS was generated and therefore a single idler could be produced.

To demonstrate the practicality of our approach for suppressing SBS, we performed a 4WM experiment using our chalcogenide chip. A CW signal with an average power of 15 mW and a pump with a peak power of about 1 W were coupled into the waveguide. As the pump power exceeded the SBS threshold, several Stokes waves were generated in the waveguide (Fig. 5a). This led to the generation of multiple idler waves via 4WM between the signal, the pump and the Stokes waves. To inhibit the generation of multiple waves at the signal and idler frequencies, we tuned the pump frequency close to the grating stopband, so that SBS was inhibited and in these conditions only a single idler and signal were produced (Fig. 5b).

## Discussion

We have demonstrated a powerful and flexible way to tailor the strength of nonlinear interactions, from strong enhancement to full suppression, at fixed input power in an on-chip highly nonlinear waveguide. Inscribing a PBG structure in the waveguides and harnessing the high optical DOS at the band edge led to a significant enhancement in SBS cascading. We applied this method to generate a Brillouin frequency comb with 15 lines in a low-Q rib waveguide. Band-edge-enhanced cascaded SBS is a promising method for the generation of on-chip coherent Brillouin frequency combs and are an attractive alternative to 4WM-based microresonator frequency combs^53^. Brillouin frequency combs exhibit GHz line spacing and can be phase locked^15^,^54^,^55^. Furthermore, SBS cascading is known to suppress the pump phase and amplitude noise with each successive Stokes component^56^. This linewidth narrowing effect is expected to play a significant role for generating low-noise Brillouin frequency combs. High spectral purity and stability are greatly desirable for on-chip microwave generation and low-noise chip-scale optical-to-digital synthesizers.

As well as enhancing nonlinear interactions, we demonstrated the feasibility of tailoring the optical DOS to fundamentally inhibit nonlinear processes. We showed that no Stokes wave was generated inside the bandgap of a PBG structure, where the DOS approaches zero. As the build-up of the Stokes wave was inhibited, no energy was transferred from the pump to the Stokes wave and, therefore, no pump depletion occurred. This technique can be exploited to improve the performance of high-power lasers and amplifiers, to design power-independent optical devices and chip-based 4WM architectures. We have demonstrated this concept in a 4WM experiment and achieved SBS-free operation. This could lead to improved communication links based on on-chip parametric amplification and all-optical signal processing schemes.

## Methods

### Experimental set-up

**Figure 6:**
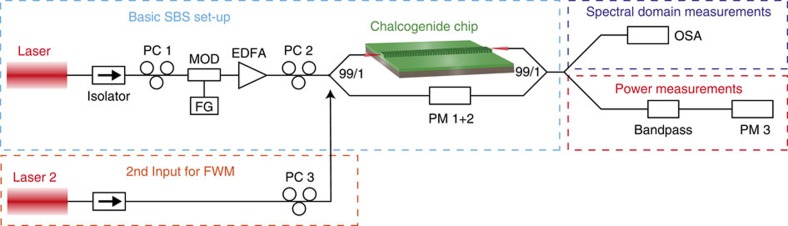
Schematic of the experimental set-up. A narrow linewidth laser was coupled to a highly nonlinear rib waveguide (light blue box). The waveguide output was monitored in the spectral domain (dark blue box) and the power domain (red box). For the 4WM experiment, a second narrow linewidth input laser was added to the set-up and coupled into the waveguide. PC, polarization controller; MOD, modulator; FG, function generator; EDFA, erbium-doped fibre amplifier; PM, power meter; OSA, optical spectrum analyser.

A schematic of the basic set-up of the experiment is shown in Fig. 6. A narrow linewidth laser at 1,550 nm with a bandwidth of 500 kHz was used as a pump source for the enhancement as well as the suppression experiments. The CW laser light was carved into 400 ns pulses with a repetition rate of 40 kHz using a Mach–Zehnder modulator driven by a function generator. The bandwidth of the coupled laser light was, therefore, determined by the pulse length and was around 2.3 MHz. The pulses were much longer than the transit time of 550 ps for the chip. Operating in a quasi-CW regime allowed high peak power levels in the waveguide while keeping the average power low. Therefore, we are able to observe strong SBS without accumulative heating of the waveguides, which prevented waveguide damage. An erbium-doped fibre amplifier was used to amplify the laser pulses before coupling to the chip, and a polarization controller (PC) allowed the coupled light to be in the transverse electric (TE) mode, which is the lowest loss mode of the waveguide. The light was coupled into the waveguide using lensed fibres with a focal spot diameter of 2 μm and a measured fibre-to-fibre coupling loss of 2.5 dB. Overall the insertion loss of the 6.8 cm long rib waveguide was 10 dB, which was permanently monitored during the experiment using a 99/1 coupler at the waveguide input and output connected to a dual-channel power meter. The refractive index of the guiding material was 2.43 leading to a maximum feedback of 17% from the cleaved facets because of the index mismatch to the surrounding air. The waveguide output was monitored with an optical spectrum analyser with 8 pm resolution.

The high-resolution spectra of the grating were measured with an optical spectrum analyser that is based on coherent detection and has a resolution of 1.2 pm. The spectra were Fourier filtered to remove the high-frequency Fabry–Perot oscillations at about 900 MHz to obtain clear spectra of the grating itself. These Fabry–Perot fringes were about 1 dB deep, as the feedback from the facets is low. Therefore, the pump and the Stokes waves did not need to be perfectly aligned and stabilized to the cavity resonances to observe SBS.

For the SBS inhibition experiment a 99/1 coupler at the waveguide output was used to simultaneously measure the pump output power (99% port) and the optical spectra (1% port). To ensure that only the pump power was measured, we used an 80 pm bandpass filter between the waveguide output and the power meter.

A second narrow linewidth laser was added to the set-up and coupled into the rib waveguide for the demonstration of SBS-free operation of a 4WM experiment (orange box in Fig. 6). Polarization controllers (PC 3 and PC 2, respectively) were used to ensure that both lasers are coupled to the TE mode of the rib waveguide.

### Sample preparation

The highly nonlinear rib waveguide was fabricated by thin-film deposition followed by several etching and annealing techniques^57^. An 850-nm-thick As_2_S_3_ layer was deposited on a thermally grown 1.5 μm thick SiO_2_ (*n*=1.44)/silicon substrate by thermal evaporation. Thermal and photo annealing of the chalcogenide thin film, led to a bulk-like refractive index of 2.43 (ref. 58). The rib waveguide was fabricated by standard contact photo-lithography and inductively coupled plasma reactive ion etching using CHF_3_ gas^59^. The etch depth for the 4 μm wide waveguides used in this work was 425 nm. As a protection layer a polytetrafluoroethylene over-cladding was deposited on the chip^60^.

The technique used for the inscription of a multi-stopband grating with the distinctive design (the stopband spacing matching the Brillouin shift) is based on the method by Hill *et al*.^33^. It utilized the photosensitivity of the As_2_S_3_ waveguide films^61^,^62^. Gratings written using this method were reported previously by us for chalcogenide fibre^34^ and the method is the same for the chip geometry.

### Simulation methods

To simulate the suppression experiment, we solved the coupled-mode equations for the pump, 1st Stokes and the acoustic wave in the presence of a PBG structure and without the PBG structure using an implicit fourth-order Runge–Kutta method^63^. The coupled-mode equations can be found in ref. 25.

For the simulation of the multi-wavelength grating, we solved the steady-state coupled equations for complex amplitudes *E*_1_ of the forward-travelling wave and *E*_2_ of the reflected backward-travelling wave, respectively, in the presence of the multibandgap structure. The equations had the following form:


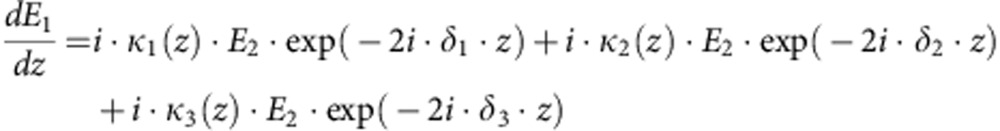



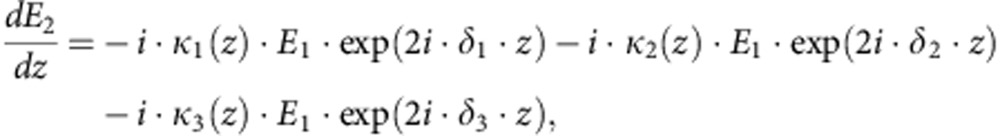


where *κ*_1_, *κ*_2_ and *κ*_3_ describe the maximum grating strength of the three stopbands and are 109, 174 and 144 m^−1^; *δ*_1_–*δ*_3_ are the detunings from the centre of the stopbands; and *z* is the spatial component along the waveguide. We solved these equations using a standard numerical ordinary differential equation solver integrating *z* over the length of the chip.

## Author contributions

M.M. conducted the experiment and the simulations under the supervision of I.V.K. and B.J.E. D.-Y.C., B.L.-D. and S.J.M. fabricated the chip. T.F.S.B. provided technical and theoretical support in the lab and for the simulations. All authors joined discussions and provided comments.

## Additional information

**How to cite this article:** Merklein, M. *et al*. Enhancing and inhibiting stimulated Brillouin scattering in photonic integrated circuits. *Nat. Commun.* 6:6396 doi: 10.1038/ncomms7396 (2015).
